# Assessment of maximal lactate steady state during treadmill exercise in SHR

**DOI:** 10.1186/1756-0500-5-661

**Published:** 2012-11-30

**Authors:** Jeeser Alves Almeida, Bernardo de Assis Petriz, Clarissa Pedrosa da Costa Gomes, Rinaldo Wellerson Pereira, Octávio Luiz Franco

**Affiliations:** 1 Centro de Análises Proteômicas e Bioquímicas, Programa de Pós-Graduação em Ciências Genômicas e Biotecnologia, Universidade Católica de Brasília, SGAN, Quadra 916, Módulo B, Av. W5 Norte, Brasília, DF, 70790-160, Brazil; 2 Programa de Pós-Graduação em Educação Física, Universidade Católica de Brasília, Brasília, DF, Brazil

**Keywords:** Hypertension, SHR, Aerobic Exercise, MLSS, Lactate, Treadmill Testing

## Abstract

**Background:**

Spontaneously hypertensive rats (SHR) are one of the main animal models used for studying the effects of exercise on hypertension. Therefore, the determination of adequate intensity has been essential for secure and optimized exercise prescriptions concerning hypertensive subjects. This study aimed to identify the MLSS in SHR by using a treadmill test to improve the protocols and further prescriptions of exercise intensity.

**Findings:**

In order to carry out this determination, SHR (n = 10) animals (~17.5 weeks; 227.4 ± 29.3 g; 172.4 ± 8.1 mmHg systolic blood pressure) were divided into two groups (G1 n = 5; G2 n = 5). Rats underwent a test with three different velocities to determine the MLSS. The MLSS was considered as the highest effort intensity where the blood lactate did not vary more than 1 mmol.L^-1^ from the 10^th^ to the 25^th^ minute. The MLSS was reached at a velocity of 20 m.min^-1^ with 3.8 ± 0.5 mmol.L^-1^ of lactate for G1. Additionally, the results were validated in G2. However, when the test was applied at 25 m.min^-1^, there was no stabilization of BLC in G1 and G2.

**Conclusions:**

In this study it was possible to identify the MLSS in SHR rats, which is an excellent evaluation tool to control exercise intensity. These data are of considerable importance in studies using physical exercise as a means of research in hypertension and may lead to the intensity of exercise being prescribed more appropriately.

## Findings

### Background

Hypertension is considered one of the main risk factors for cardiovascular disease and may be closely associated with other pathologies such as diabetes and obesity [1]. Several pathophysiologic factors may lead to hypertension, such as a stressful lifestyle related to increased sympathetic nervous system activity. Furthermore, high sodium consumption and hyper caloric diets may also lead to hypertension and to diabetes mellitus, obesity and perturbation of the renin-angiotensin-aldosterone system, impairing vascular tone and increasing endothelial dysfunction [2]. Therefore, independently of hypertension pathogenesis, its chronic maintenance is itself a strong factor of cardiovascular diseases, leading to high death rates worldwide [1].

Along with dietary and pharmacological treatment, chronic exercise stimulus has been recommended as an alternative and effective way to prevent and treat hypertension, since it reduces resting blood pressure rates [3]. As well as chronic stimulus, blood pressure (BP) is also decreased at resting rates after one single session of exercise, a process known as post exercise hypotension (PEH), which started to attract clinical attention at the beginning of the 1980′s [4]. The PEH phenomenon has been widely researched under the influence of aerobic exercise and to a lesser extent under resistance training [5]. However, there are concerns about the adequate intensity and duration of exercise that may lead to a potential hypotension effect. The determination of adequate intensity is therefore essential for secure and optimized exercise prescriptions involving hypertensive subjects [5]. To this end, animal models are often used in exercise research, especially to establish adequate training exercise intensities. Spontaneously hypertensive rats (SHRs) are one of the main animal models used to verify the positive effects of exercise in the hypertensive phenotype, as well as to understand the biologic dysfunctions caused by this pathology [6]. Nevertheless, there is a lack of methodological data that serve to establish adequate exercise intensity in hypertensive animal models. Blood lactate concentration (BLC) is a valuable tool in determining the intensity of exercise and is commonly accepted as a performance index [7]. Therefore, the maximal lactate steady state (MLSS) is considered the gold standard in assessing aerobic capacity, since it is based on the identification of the greatest intensity of exercise in which there is no increase in lactate production [8]. This represents the time when the production/removal of lactate is in equilibrium [9]. The MLSS has been identified in different animal models [10,11], such as in rats swimming or using a treadmill in sedentary, trained, adult and old animals [12-14]. However, until now, the MLSS protocol has not been identified in hypertensive animal models such as SHR. Consequently, this study aims to identify the MLSS during treadmill exercise in hypertensive rats in order to further improve protocols, and prescription of exercise intensity training for hypertensive subjects. Moreover, the relationship between aerobic power and MLSS in SHR was also verified.

### Materials and methods

#### Animals

Ten female spontaneously hypertensive rats (~17.5 weeks; 227.4 ± 29.3 g; 172.4 ± 8.1 mmHg systolic blood pressure) were used. These isogenic animals were obtained from the bioterium of the Federal University of São Paulo, Brazil. Water and food were provided *ad libitum*, and the animals were kept in a 12:12 h dark–light cycle in a room at 23 ± 2°C. The study was approved by the ethics committee on animal use of the University of Brasilia, Brazil. All procedures were in accordance with the Brazillian College of Animal Experimentation (COBEA) [15].

#### Exercise procedures and MLSS

Only animals showing physical fitness in treadmill exercise were chosen (n = 10). All animals underwent treadmill (Li 870, Letica Scientific Instruments, Barcelona, Spain) adaptation according to Contarteze *et al*. [12]. After the adaptation period (5 days/week for three weeks, where environmental adaptation, duration and velocity were progressively increased), a group of rats (G1, n = 5) underwent a test at three different velocities to determine the MLSS (15 m.min^-1^, 20 m.min^-1^ and 25 m.min^-1^). The velocities were set randomly, and the tests were carried out with a 48 h interval between them. The tests lasted for 25 min of continuous exercise (0% graded) or until animal exhaustion. Capillary blood was collected every 5 min from the distal portion of the tail of animals for BLC analysis. This protocol has been previously applied in Wistar rats [12]. The MLSS was considered as the highest intensity of effort where the blood lactate did not vary more than 1 mmol.L^-1^ from the 10^th^ to the 25^th^ min [12].

#### Incremental test

Additionally, a maximal incremental test (IT) was performed with 0% graded exercise with increments of 3 m.min^-1^ every 3 min [16], starting at 8 m.min^-1^ until animal exhaustion. In order to verify the reliability of results obtained in G1, the same procedures were applied to an extra group of SHRs (G2) (n = 5).

#### Blood collection and analysis

From a small incision in the distal tail portion, 10 μL of blood was collected in capillaries, rapidly deposited in microtubes (0.6 mL) containing 20 μL of 1% sodium fluoride and stored at −20°C for further biochemical analysis. The electro-enzymatic method from YSI Sports (Yellow Springs, OH, USA) was utilized for BLC analyses [12].

#### Statistical analysis

All analyzed data were normally distributed and a Kolmogorov-Smirnov test was applied to verify the normality of data present (p > 0.10). The data are presented as mean ± SD, and statistical analyses consisted of one-way ANOVA for repeated measures with Bonferroni post-hoc test. The level of significance was set at p < 0.05.

### Results

**Figure 1:**
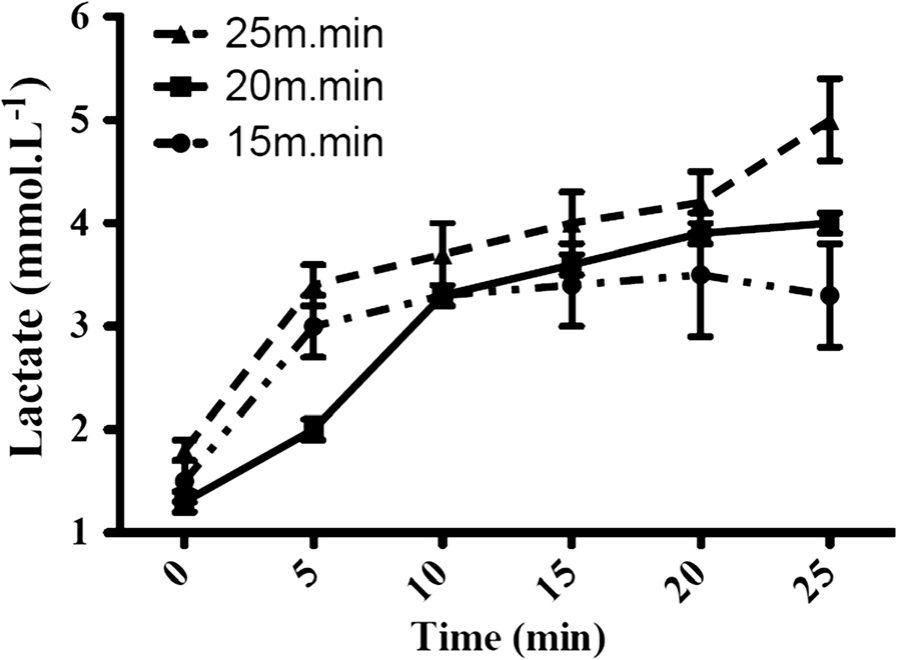
**Blood lactate concentration (mean ± SD) during the identification of the MLSS in treadmill running test at 15 m.min**^**-1**^**, 20 m.min**^**-1**^**and 25 m.min**^**-1 **^**velocities in G1.** Vertical bars correspond to standard deviation.

The G1 results showed stabilization of BLC at 15 m.min^-1^. Otherwise, when applied at 20 m.min^-1^, stabilization of BLC occurred contrary to the test performed at 25 m.min^-1^, which showed an increase of BLC. Therefore, when the test was applied at 20 m.min^-1^, it was possible to identify the MLSS, as shown in Figure 1. Aiming to confirm the data obtained from G1, the same procedures were applied to G2. Similar results were obtained from G2, and stabilization of BLC occurred at the velocity of 15 m.min^-1^ and 20 m.min^-1^. However, when the velocity of 25 m.min^-1^ was applied, the BLC did not show stable behavior. Thus, the MLSS results found in G1 and G2 (20 m.min^-1^) showed no statistical differences (p > 0.05) (Table 1). Therefore, the MLSS was found at the velocity of 20 m.min^-1^ in both groups. By the incremental test (IT), animals showed similar results, reaching maximum velocity of 27 ± 2.7 m.min^-1^ for G1 and 26.5 ± 2.2 m.min^-1^ for G2 (P > 0.05). The MLSS identified in this study for SHRs represents approximately ~75% of the maximum velocity achieved during the incremental test.

**Table 1:** Blood lactate concentration (mean ± SD) after running on a treadmill at different velocities for G1 and G2 groups

	**15 m.min**^**-1**^	**20 m.min**^**-1**^	**25 m.min**^**-1**^
**G1**	**3.3**	**3.8**	**4.9**
	*0.1*	*0.3*	*0.5*
**G2**	**3.4**	**3.9**	**5.1**
	*0.4*	*0.1*	*0.2*

ANOVA showed no statistically significant differences (G1 vs. G2) (P > 0.05).

### Discussion

The present study aimed to identify the MLSS in rats of the SHR strain, an important animal model in studies involving the understanding of hypertension. As far as we know, this is the first MLSS identification in SHR animals, setting their aerobic capacity in a treadmill device at the velocity of 20 m.min^-1^ in G1 with blood lactate concentration of 3.8 ± 0.3 mmol.L^-1^. These data reinforce the role of proper exercise intensity for attenuating physiological dysfunctions caused by hypertension. The increasing number of studies related to the benefits of exercise for hypertensive phenotype led exercise to be widely recommended as a non-pharmacological treatment [17] for hypertension attenuation. However, the precise prescription of exercise should be taken into consideration, especially regarding the intensity of exercise. Considering that one of the benefits of exercise for hypertensive subjects is the PEH effect [3,18], and since exercise intensity seems to influence the magnitude of the PEH effect [19,20] it is particularly important to establish the appropriate intensity at which the exercise is carried out, With this in mind, Lee *et al.*[21] showed a PEH in SHR (15 m.min^-1^ for 20 min), and there are several other studies that showed substantial benefits of exercise in SHR model [22-24]. Furthermore, it has been observed that hypertensive myocardium improved after 12 weeks of running (60 min, 5 days/week) at the velocity of 20–25 m.min^-1^[6], which corresponds to exercise performed both above and at the MLSS intensity determined by this study.

The MLSS is commonly considered the gold standard for aerobic capacity determination. Nevertheless, other methods can also be applied to identify the anaerobic threshold, which similarly could be used for aerobic capacity determination [25]. In an attempt to determine aerobic capacity in Wistar rats, Contarteze *et al.*[12] found an intensity corresponding to lactate threshold at 20 m.min^-1^ with a BLC of 3.8 ± 0.1 mmol.L^-1^. Interestingly, in our study we also found the aerobic capacity at 20 m.min^-1^ with a BLC of 3.8 ± 0.3 mmol.L^-1^ using the gold standard method (MLSS) (Figure 1). Although Wistars and SHRs exhibit the same aerobic capacity, Ceroni *et al.*[26], showed that SHR that underwent an IT revealed higher performance (aerobic power) when compared to Wistar rats. IT is widely used to prescribe the intensity of exercise [27], since it is easier to apply than the procedure for determining the MLSS, which requires more time to perform than the anaerobic threshold determination by ITs. In Wistar rats, this could be justified by the fact that usually the lactate threshold identified by an IT does not differ from MLSS [14]. However, the MLSS for determination of the aerobic capacity had not yet been identified in the SHR to date, and this study is the first to identify this MLSS in SHR during treadmill running. Additionally, the MLSS speed in this study represents ~75% of aerobic power determined by IT, which makes it clear that hypertensive rats exhibit excellent aerobic capacity, as previously observed by Ceroni *et al*. (2009). However, it is important to remember that there are several study limitations. Among them, anaerobic threshold and VO_2max_ were not identified. To this end, further studies must be performed in order to identify these variables and confirm our findings. Such data could improve the training prescription standards in SHR animal model.

### Conclusions

The results of this study provide an appropriate way to determine the intensity of physical exercise in hypertensive rats, so that future studies can use the MLSS as a tool to evaluate aerobic capacity, appropriate physical training intensity prescription and validate other protocols of physical assessment. However, although this study was the first to identify the MLSS in SHR on a treadmill, the lactate threshold was not identified by IT. Since the SHR is an important model for studying diseases such as arterial hypertension, the use of MLSS is recommended in future studies, in order to ensure more accurate results with respect to exercise.

## Abbreviations

SHR: Spontaneously Hypertensive Rats; MLSS: Maximal Lactate Steady State; BLC: Blood lactate concentration; SHHF: Spontaneously hypertensive heart failure; PEH: Post exercise hypotension; BP: Blood pressure.

## Competing interests

The author(s) declare that they have no competing interests.

## Authors’ contributions

JAA; carried out the study design, performed the animal training and statistical analysis and drafted the manuscript. BAP; carried out the study design, performed the animal training and drafted the manuscript. CPCG; carried out the study design, performed the animal training and drafted the manuscript. RWP; carried out the study design and drafted the manuscript. OLF; carried out the study design and revised the manuscript. All authors read and approved the final manuscript.

## References

[B1] Sliwa K Stewart S Gersh BJ Hypertension: a global perspective Circulation 2011 123 2892 2896 10.1161/CIRCULATIONAHA.110.992362 21690504

[B2] Oparil S Zaman MA Calhoun DA Pathogenesis of hypertension Ann Intern Med 2003 139 761 776 14597461 10.7326/0003-4819-139-9-200311040-00011

[B3] MacDonald JR Potential causes, mechanisms, and implications of post exercise hypotension J Hum Hypertens 2002 16 225 236 10.1038/sj.jhh.1001377 11967715

[B4] Fitzgerald W Labile hypertension and jogging: new diagnostic tool or spurious discovery? Br Med J (Clin Res Ed) 1981 282 542 544 10.1136/bmj.282.6263.542 PMC1504300 6780119

[B5] Anunciação PG Polito MD A Review on Post-exercise Hypotension in Hypertensive Individual Arq Bras Cardiol 2011 96 425 426 10.1590/S0066-782X2011005000025 21359479

[B6] Kolwicz SC MacDonnell SM Renna BF Reger PO Seqqat R Rafiq K Kendrick ZV Houser SR Sabri A Libonati JR Left ventricular remodeling with exercise in hypertension Am J Physiol Heart Circ Physiol 2009 297 H1361 H1368 10.1152/ajpheart.01253.2008 19666835 PMC2770762

[B7] Beneke R Leithauser RM Ochentel O Blood lactate diagnostics in exercise testing and training Int J Sports Physiol Perform 2011 6 8 24 21487146 10.1123/ijspp.6.1.8

[B8] Beneke R von Duvillard SP Determination of maximal lactate steady state response in selected sports events Med Sci Sports Exerc 1996 28 241 246 10.1097/00005768-199602000-00013 8775160

[B9] Billat VL Sirvent P Py G Koralsztein JP Mercier J The concept of maximal lactate steady state: a bridge between biochemistry, physiology and sport science Sports Med 2003 33 407 426 10.2165/00007256-200333060-00003 12744715

[B10] Lindner AE Maximal lactate steady state during exercise in blood of horses J Anim Sci 2010 88 2038 2044 10.2527/jas.2009-2693 20190168

[B11] Ferreira JC Rolim NP Bartholomeu JB Gobatto CA Kokubun E Brum PC Maximal lactate steady state in running mice: effect of exercise training Clin Exp Pharmacol Physiol 2007 34 760 765 10.1111/j.1440-1681.2007.04635.x 17600553

[B12] Contarteze RV Manchado FB Gobatto CA De Mello MA Stress biomarkers in rats submitted to swimming and treadmill running exercises Comp Biochem Physiol A Mol Integr Physiol 2008 151 415 422 10.1016/j.cbpa.2007.03.005 17428717

[B13] Manchado FB Gobatto CA Contartaze RVL Papoti M Mello MAR Maximal lactate steady state in running rats J Exerc Physiol 2005 8 29 35

[B14] Cunha RR Cunha VN Segundo PR Moreira SR Kokubun E Campbell CS de Oliveira RJ Simoes HG Determination of the lactate threshold and maximal blood lactate steady state intensity in aged rats Cell Biochem Funct 2009 27 351 357 10.1002/cbf.1580 19585487

[B15] Harriss DJ Atkinson G Update–Ethical standards in sport and exercise science research Int J Sports Med 2011 32 819 821 22065312 10.1055/s-0031-1287829

[B16] Rodrigues B Figueroa DM Mostarda CT Heeren MV Irigoyen MC De Angelis K Maximal exercise test is a useful method for physical capacity and oxygen consumption determination in streptozotocin-diabetic rats Cardiovasc Diabetol 2007 6 38 10.1186/1475-2840-6-38 18078520 PMC2222609

[B17] Wallace JP Exercise in hypertension. A clinical review Sports Med 2003 33 585 598 10.2165/00007256-200333080-00004 12797840

[B18] Mach C Foster C Brice G Mikat RP Porcari JP Effect of exercise duration on postexercise hypotension J Cardiopulm Rehabil 2005 25 366 369 10.1097/00008483-200511000-00010 16327532

[B19] Jones H George K Edwards B Atkinson G Is the magnitude of acute post-exercise hypotension mediated by exercise intensity or total work done Eur J Appl Physiol 2007 102 33 40 10.1007/s00421-007-0562-0 17879098

[B20] Morais PK Campbell CS Sales MM Motta DF Moreira SR Cunha VN Benford RE Simões HG Acute resistance exercise is more effective than aerobic exercise for 24 h blood pressure control in type 2 diabetics Diabetes Metab 2011 37 112 117 10.1016/j.diabet.2010.08.008 21159536

[B21] Lee SK Kim CS Kim HS Cho EJ Joo HK Lee JY Lee EJ Park JB Jeon BH Endothelial nitric oxide synthase activation contributes to post-exercise hypotension in spontaneously hypertensive rats Biochem Biophys Res Commun 2009 382 711 714 10.1016/j.bbrc.2009.03.090 19306842

[B22] Garciarena CD Pinilla OA Nolly MB Laguens RP Escudero EM Cingolani HE Ennis IL Endurance training in the spontaneously hypertensive rat: conversion of pathological into physiological cardiac hypertrophy Hypertension 2009 53 708 714 10.1161/HYPERTENSIONAHA.108.126805 19221208

[B23] Melo RM Martinho E Jr Michelini LC Training-induced, pressure-lowering effect in SHR: wide effects on circulatory profile of exercised and nonexercised muscles Hypertension 2003 42 851 857 10.1161/01.HYP.0000086201.27420.33 12913057

[B24] Sun MW Qian FL Wang J Tao T Guo J Wang L Lu AY Chen H Low-intensity voluntary running lowers blood pressure with simultaneous improvement in endothelium-dependent vasodilatation and insulin sensitivity in aged spontaneously hypertensive rats Hypertens Res 2008 31 543 552 10.1291/hypres.31.543 18497475

[B25] Pardono E Sotero Rda C Hiyane W Mota MR Campbell CS Nakamura FY Simoes HG Maximal lactate steady-state prediction through quadratic modeling of selected stages of the lactate minimum test J Strength Cond Res 2008 22 1073 1080 10.1519/JSC.0b013e318173c594 18545205

[B26] Ceroni A Chaar LJ Bombein RL Michelini LC Chronic absence of baroreceptor inputs prevents training-induced cardiovascular adjustments in normotensive and spontaneously hypertensive rats Exp Physiol 2009 94 630 640 10.1113/expphysiol.2008.046128 19251981

[B27] Felix JV Michelini LC Training-induced pressure fall in spontaneously hypertensive rats is associated with reduced angiotensinogen mRNA expression within the nucleus tractus solitarii Hypertension 2007 50 780 785 10.1161/HYPERTENSIONAHA.107.094474 17646572

